# Dusting utilizing suction technique (DUST) for percutaneous nephrolithotomy: use of a dedicated laser handpiece to treat a staghorn stone

**DOI:** 10.1590/S1677-5538.IBJU.2017.0266

**Published:** 2018

**Authors:** Khurshid R. Ghani, Ali H. Aldoukhi, William W. Roberts

**Affiliations:** 1 Department of Surgery, VA Ann Arbor Healthcare System, Ann Arbor, MI, USA; 2 Department of Urology, University of Michigan, Ann Arbor, MI, USA

## Abstract

**Introduction:**

Dusting, use of high frequency and low pulse energy-is commonly performed during ureteroscopic holmium laser lithotripsy but reports on the ability of this method to treat large stones via percutaneous nephrolithotomy (PCNL) are limited. We report on the first clinical feasibility of a dusting technique during PCNL using a specially designed laser suction handpiece (LSHP).

**Materials and Methods:**

We performed PCNL on a patient with spinal cord injury, and recurrent urinary tract infection. Computed tomography demonstrated a left complete staghorn stone, 1000 Hounsfield units. Standard (30F) prone PCNL was performed via lower pole access and balloon dilatation. A 120-Watt holmium (P120H, Lumenis) was used to perform Dusting Utilizing a Suction Technique (D.U.S.T.) for PCNL with a 550um fiber in the LSHP which was connected to a suction pump in the P120H. The LSHP has a stainless steel cannula with inner lumen diameter of 3.25mm, with fiber positioned in a separate working channel on top of the cannula. Suction is activated via the LSHP, and fragments are sucked into a collection container. We used it in “automatic” mode where suction occurred only during laser activation.

**Results:**

We successfully performed DUST-PCNL to treat the staghorn stone using settings of 0.6Jx70Hz, and 1.0Jx60Hz, (long pulse width). Total operative time was 110 minutes; laser time 21.29 minutes, laser energy 47.68kJ. We did not encounter any difficulty with fragment aspiration or clogging of the cannula or suction tubing. Ancillary devices used included a basket to retrieve large fragments, and flexible nephroscopy to dust an upper pole branch of the staghorn stone. A nephrostogram on post-operative day POD 1 demonstrated a 4mm residual fragment. Patient was discharged on POD 2. There were no complications; stone analysis demonstrated a struvite stone.

**Conclusions:**

We confirmed initial clinical feasibility and safety of DUST-PCNL to perform simultaneous lithotripsy and aspiration for effective stone clearance. An advantage of this method is versatility in treating a stone with both rigid and flexible endoscopy using a single energy source. Further evaluation is needed to better understand the efficacy of this technique.

## ARTICLE INFO

**Available at: http://www.intbrazjurol.com.br/video-section/20170266_Ghani_et_al**

**Int Braz J Urol. 2018; 44 (Video #10): 840-1**

